# Temporal activity patterns of North China leopards and their prey in response to moonlight and habitat factors

**DOI:** 10.1002/ece3.9032

**Published:** 2022-06-23

**Authors:** Muhammad Zaman, Nathan James Roberts, Mengyan Zhu, Kasereka Vitekere, Meng Wang, Guangshun Jiang

**Affiliations:** ^1^ Feline Research Center of China National Forestry and Grassland Administration, College of Wildlife and Protected Area Northeast Forestry University Harbin China; ^2^ College of Life Science Yanan University Yanan China; ^3^ University of Goma Goma Democratic Republic of Congo; ^4^ Chengdu Zoo Chengdu China

**Keywords:** Camera trap, habitat factors, moon phase, North China leopard, prey, temporal overlap

## Abstract

The nocturnal activities of predators and prey are influenced by several factors, including physiological adaptations, habitat quality and, we suspect, corresponds to changes in brightness of moonlight according to moon phase. In this study, we used a dataset from 102 camera traps to explore which factors are related to the activity pattern of North China leopards (*Panthera pardus japonensis*) in Shanxi Tieqiaoshan Provincial Nature Reserve (TPNR), China. We found that nocturnal activities of leopards were irregular during four different lunar phases, and while not strictly lunar philic or lunar phobic, their temporal activity was highest during the brighter moon phases (especially the last quarter) and lower during the new moon phase. On the contrary, roe deer (*Capreolus pygargus*) exhibited lunar philic activity, while wild boar (*Sus scrofa*) and tolai hare (*Lepus tolai*) were evidently lunar phobic, with high and low temporal activity during the full moon, respectively. In terms of temporal overlap, there was positive overlap between leopards and their prey species, including roe deer and tolai hare, while leopard activity did not dip to the same low level of wild boar during the full moon phase. Human activities also more influenced the temporal activity of leopards and wild boar than other species investigated. Generally, our results suggested that besides moonlight risk index (MRI), cloud cover and season have diverse effects on leopard and prey nocturnal activity. Finally, distinct daytime and nighttime habitats were identified, with leopards, wild boar, and tolai hare all using lower elevations at night and higher elevations during the day, while leopards and roe deer were closer to secondary roads during the day than at night.

## Graphical abstract

we used a dataset from 102 camera traps to explore which factors are related to the activity pattern of North China leopards (Panthera pardus japonensis) in Shanxi Tieqiaoshan Provincial Nature Reserve (TPNR), China. Finally, recognizing that the North China Leopard (P. p. japonensis) is a critically endangered endemic species, to aid the conservation and management of this predator and its prey

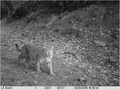

## 1 INTRODUCTION

Worldwide, 69% of mammals are nocturnal, while 20% are exhibiting more diurnal activity patterns (Bennie et al., 2014), and this phenomenon is an ancestral character stemming from the “nocturnal bottleneck” in the early evolution of the clade (Hut et al., 2012). Although synapsids invaded the nocturnal niche 100 million years prior to mammals, recent studies support the essential nocturnality of ancestral mammals, by way of selection for dim‐light vision (“night vision”), endothermy, and loss of UV protection (Angielczyk & Schmitz, 2014; Wu et al., 2017).

The familiar cycle of lunar phases is recognized by variability in the visible portion of the moon illuminated by the sun (Andreatta & Tessmar‐Raible, 2020), with the intensity of lunar illumination on the Earth's surface at night varying by three orders of magnitude over a full lunar cycle (Kyba et al., 2017). Moreover, other factors such as topography, cloud cover, latitude, and distance from the moon, each play a role in influencing the intensity of lunar illumination in any given space. Nocturnal organisms can react directly to changes in lunar illumination as the moon cycles through the phases, and they can also anticipate variations that go along with the lunar cycle by means of an endogenous oscillator (“clock”) synchronized to the 29.5‐day circalunar rhythm (Raible et al., 2017).

The primary environmental cue that changes with the lunar cycle is moonlight intensity (Andreatta & Tessmar‐Raible, 2020; Häfker & Tessmar‐Raible, 2020), and these cues (or “zeitgebers”) act on endogenous oscillators to adjust biological processes such as mating, feeding, activity, predator avoidance, and many others (Andreatta & Tessmar‐Raible, 2020). The idea that being afraid of the dark is an adaptation to dodge predation has a long history (Darwin, 1871), and the moonlight cycle as a cue for predation risk was first studied in nocturnal desert rodents several decades ago (Lockard & Owings, 1974).

Animals' circadian activity patterns are influenced by evolution (Halle, 2000) and physiology (Heurich et al., 2014), and we find that prey species with powerful tapeta (and therefore, have superior night vision) tend to be lunar philic (light lovers), whereas species with deficient tapetum lucidum (i.e., have poor night vision) are more lunar phobic (light avoiding) or uninfluenced by lunar phase (Prugh & Golden, 2014). According to the “predation risk hypothesis” (Huck et al., 2017), if predators are more successful at chasing under bright moonlight, prey species will alter the activity to become “lunar phobic” or avoid brighter moon phases. On the contrary, the “visual acuity hypothesis” (Huck et al., 2017; Pratas‐Santiago et al., 2017) states that the brightness of a full moon provides “visually oriented” prey species heightened chance to forage and/or detect danger, with the result that they are expected to be more active during the full moon, showing “lunar philic” activity—in other words preferring brighter moon phases (Fernández Moya et al., 2021). For example, brighter nights seem to inhibit the activity of ocelots (*Leopardus pardalis*) (Leonard et al., 2020), two‐toed sloth (*Choloepus didactylus*) (De Miranda et al., 2020), and red muntjac (*Muntiacus muntjak*) (Rahman & Mardiastuti, 2021), while for other species, such as cheetah (*Acinonyx jubatus)* (Broekhuis et al., 2014), bobcats (*Lynx rufus*) (Leonard et al., 2020), Bawean deer *(Axis kuhlii*) (Rahman & Mardiastuti, 2021), Indian hare *(Lepus nigricollis)* (Bhatt et al., 2021), and some nocturnal birds (Pérez‐Granados & Schuchmann, 2021), brighter nights yield increased activity.

Besides lunar illumination, the activity patterns of large carnivores, such as felids, are also influenced by anthropogenic disturbances (Gaynor et al., 2018; Van Cleave et al., 2018). For example, studies have shown that leopards (*Panthera pardus*) in Thailand (Ngoprasert et al., 2007) and Amur tigers (*Panthera tigris altaica*), and leopards (*Panthera pardus orientalis*) in China (Yang, Zhao, et al., 2018; Zhao et al., 2020) shift their activity patterns under the effect of human disturbance to become more nocturnal or crepuscular and avoid human activity across spatiotemporal scales to decrease the risk of conflict with humans (Treves & Karanth, 2003; Wang et al., 2019).

Furthermore, the landscape of fear created by predators may be linked to the spatial distribution of prey and changes in temporal foraging movements (Bischof et al., 2014). Further to temporal activity patterns, animals may also alter habitat resource selection in different seasons (Ramesh et al., 2012; Yang, Zhao, et al., 2018); hence, spatial separation is another strategy for the coexistence of sympatric species (Davis et al., 2018; Yang, Zhao, et al., 2018; Zhao et al., 2020).

The North China leopard (*P. p. japonensis*), hereafter leopard, has a distribution and ecological knowledge base which is very limited and has been classified as Critically Endangered on the IUCN Red List (Jacobson et al., 2016). Basic information about how various ecological factors influence leopard and prey species co‐occurrence under different lunar phases is still unknown in China, and the current research aims to fill this gap.

The aims of the current study were to examine the temporal activity patterns of leopards and prey species, as well as the effects of human‐related activities, habitat factors, and lunar illumination. Specifically, we tested the following three hypotheses: (1) the effects of different moon phases on nocturnal spatiotemporal overlap intensity will be distinct among different predator–prey pairs, and we predict that more intense moonlight will increase predation risk by enhancing the ability of predators to detect prey (Bhatt et al., 2021), thus leading to decreased activity or shifts in prey foraging efficiency in brighter nights, or prey will shift their activity to other moon phases or daytime (Botts et al., 2020); (2) habitat factors influence the activity response of prey to leopards during the four moon phases, and we assume that the apex predator controls spatial distribution and temporal activity of preys with respect to habitat preference or avoidance (Zhu et al., 2021), while cloud cover may also influence spatiotemporal distribution (Botts et al., 2020); (3) predator and prey species shift their temporal activity patterns during the full moon such that higher activity during the day time counters the brightness of the moon at night time. We assumed that most cats are nocturnal (Bhatt et al., 2021) or crepuscular, and that they hunt in the dark (Fernández Moya et al., 2021). Hence, it may be logical to assume that the lunar phase could also affect hunting success and influence prey to reduce their nighttime activity and shift to daytime to reduce predatory attacks (Yang et al., 2021). We also expect that predator–prey daytime activity may be influenced by human activities on foot occurring at lower elevations (Qi et al., 2022), and that low human activity at nighttime can drive the activity of predators and prey at low elevation (Zhu et al., 2021), while also avoiding human settlements (Lamichhane et al., 2019; Qi et al., 2022).

## 2 MATERIALS AND METHODS

### 2.1 Study area

**FIGURE 1:**
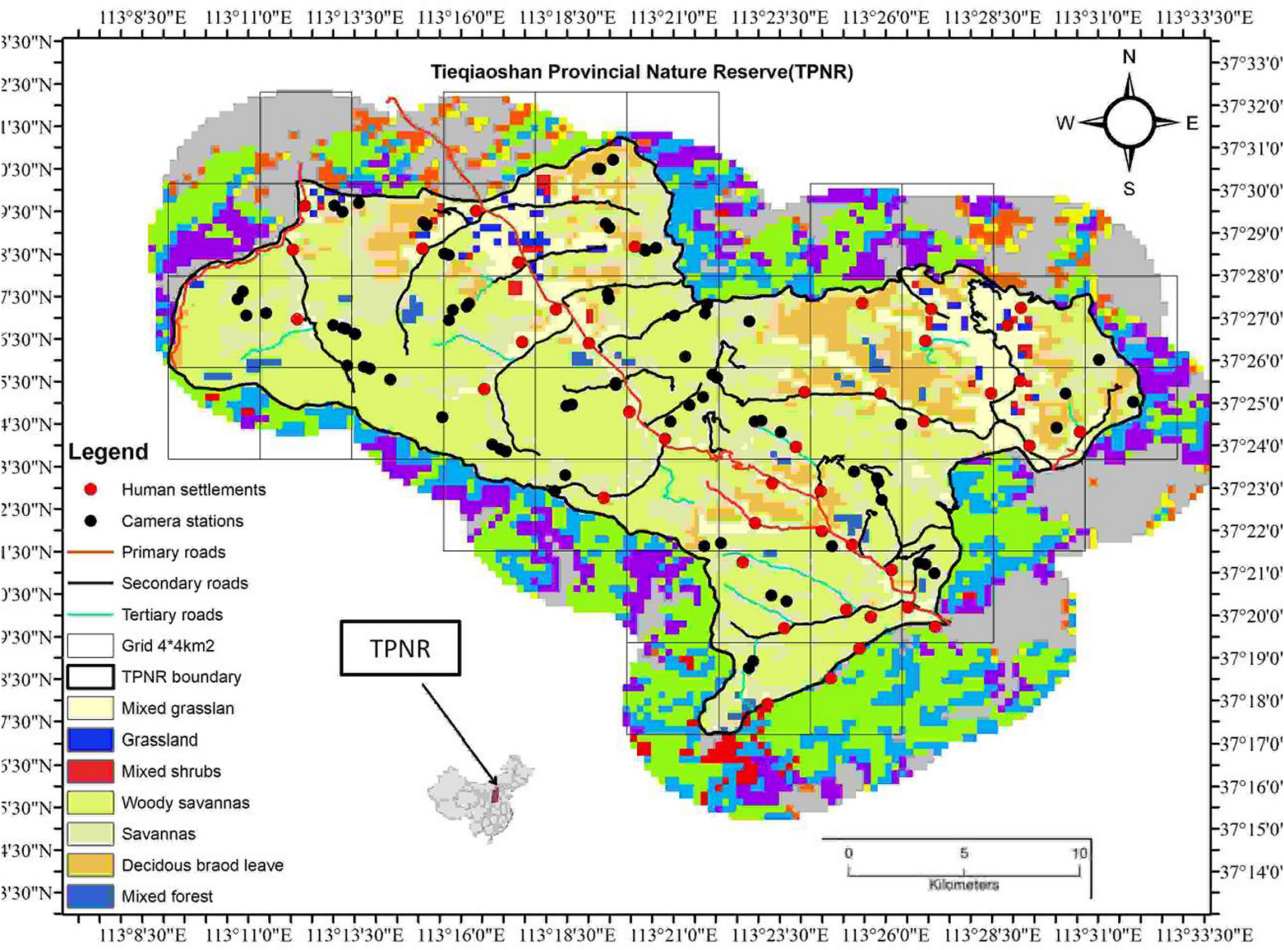
Map of study area showing type of roads, human settlements, and camera points. TPNR represents the Tieqiaoshan Provincial Nature Reserve, Shanxi, and China. Shading outside the TPNR boundary represents habitat structure in the surrounding area, but is not defined here

This study was conducted in Tieqiaoshan Provincial Nature Reserve (TPNR) in Shanxi Province, China (Figure 1). The TPNR is a federally protected area established in 2002 by the federal people's administration, permitting local population settlement in the TPNR with strict actions of wildlife protection. The altitude ranges between 1400 m and 1700 m a.s.l. The total protected area of the reserve is 35,351.7 ha, with a core zone area of 13,948.6 ha and a buffer area of 7401.7 ha. There are 40 villages located in the buffer zone of Tie Qiao Forest Bureau with approximately 30,022 inhabitants; the density is 85 inhabitants/km^2.^ The majority of people practice Buddhism and their livelihood is mainly based on agricultural crops and livestock (goat *Capra hircus*, sheep *Ovis aries*, and cow *Bos indicus*), along with pets or stray dogs which are found in each village. Typically, anthropogenic activities occur during the daytime in the low elevation area of the buffer zone and rarely in the core zone around secondary and tertiary roads (Zhu et al., 2021). The climate is warming temperate in summer and continental in winter. The annual rainfall is about 600 mm and the average annual temperature is about 6°C, while the daily average temperature recorded in this area is above 10°C (Zhu et al., 2021).

The area also has high wildlife diversity, containing 24 species of mammals, 6 species of reptiles, 3 species of amphibians, and 116 species of birds. The flora is a mix of deciduous broadleaf, coniferous, and mixed deciduous forests. The common tree species are Chinese red pine (*Pinus tabuliformis*), Liaotung oak (*Quercus liaotungensis*), white birch (*Betula platyphylla*), and larch (*Larix principis*). Rarer trees include willow (*Salix spp*), apricots (*Prunus spp*.), etc., and dominant shrubs include sea buckthorn, (*Hippophae spp*.), *Berberis spp*., and *Artemisia* spp. Overall, the TPNR has 78 genera of wild seed plants (Hua et al., 2020). Major threats for this reserve are tree logging at the forest edge, overgrazing, forest fire, and human‐carnivore conflicts (Tieliang, 1985).

### 2.2 Data collection

#### 2.2.1 Camera trapping videos and photos, including pre‐processing

Initially, the whole area was divided into a grid system of size 4 × 4 km (Figure 1) and between two and five camera‐trap locations in each grid cell were selected based on the presence of carnivore signs and prey trails. However, finally, the camera trap array was less systematic as some grid cells were inaccessible for our team, while some grid cells had additional cameras installed where there were relatively high occurrences of leopards and prey; this protocol follows Zhu et al. (2021).

Two infrared cameras (Eastern Red Hawk E1B 6210 M and LTL6210MM, Manufactured by Shenzhen Weixin Science and Technology Development Co. Ltd. Shenzhen, China) were set facing each other at each trap station to increase capture probabilities and capture the fur patterns on both sides of leopards; one camera was set to record short videos (10 second length), and the other camera recorded photos (three photos per trigger event). In order to capture quality images, cameras were attached to trees 45–50 cm above the ground at a distance of 3.5–4 m from animal trails, and all vegetation or other obstacles in front of the cameras were removed (Qi et al., 2015). All photographs were automatically stamped with the time and date, moon phase, and respective location ID. A total of 102 camera locations operated between March 2017 and June 2019, and among them, 78 cameras successfully functioned to collect data over three sampling stages in two different seasons (see below for details) with 131 (March–July 2017), 120 (September–December 2018), and 134 (March–June 2019) consecutive days being sampled. Each camera was visited approximately every 2 months to download image files and check/replace batteries. The remaining 24 cameras were stolen or damaged during the study period.

The 24‐h activity patterns of all species were deduced from camera‐trap photo and video records (Qi et al., 2015; Yang, Zhao, et al., 2018). We analyzed only photos taken at a minimum time interval of 30 min (Santos et al., 2019) to avoid pseudoreplication. Based on local climate characteristics, we defined two distinct periods: the winter period (snow period: November–April) and the summer period (snow‐free period: May–October) (Table 1). The data processing was completed by specialists in Prof. Jiang Guangshun's research teams at the Feline Research Center of National Forestry and Grassland Administration (Northeast Forestry University) who completed species identification and data arrangement. Note that in our analyses, as we did not conduct diet analyses, we referred to literature to determine potential prey species of leopards (Table S1) including Sri Lankan leopard (*P. p. kotiya)* (Kittle et al., 2014), Indian leopards *(P. p.fusca)* (Desai et al., 2021; Kshettry et al., 2018), Amur leopards (*p. p. orientalis*) (Yang, Dou, et al., 2018), Persian leopards *(P. p. saxicolor)* (Sharbafi et al., 2016), Arabian leopard (*P. p. Nimr*) (Stuart, 2017), Indochinese leopards (*P. p. delacouri*) (Drew, 2000; Stuart & Stuart, 2007), snow leopard (*Panthera uncial*) (Khatoon et al., 2017), North China leopard (Yang et al., 2021), African leopard (*P. p. pardus*) (Havmøller et al., 2021), Javan leopard *(P. p. melas)* (Rahman et al., 2018).

**TABLE 1:** Habitat variables used for the generalized linear mixed models (GLMMs) to model the drivers of leopard and prey species daily activity

Variable	Description	Data type	Unit/source
Habitat factors	Records were taken from the National Geomatics Center of China (http://www.ngcc.cn/ngcc/)		
Deciduous forest	Distance to the edge of the nearest deciduous broadleaf forest (oak)	Continuous	(m)
Mixed forest	Distance to the edge of nearest mixed forest	Continuous	(m)
Woody savannas	Distance to the edge of nearest woody savanna (pine tree)	Continuous	(m)
Grassland	Distance to the edge of nearest grassland	Continuous	(m)
Elevation	Elevation at each camera station as described by Qi et al. (2015)	Continuous	(m) Field data
Season	Summer or winter period of capture event, determined by the time stamp on camera trap image/video; summer = 1, winter = 0	Binary	Camera trap
Activity times	24 h diel activities of animals in each season calculated using independent photos as described by Zhao et al. (2020)	Continuous	Moonrise
Clouds	Clear and overcast weather during capture event; 0 = overcast, and 1 = clear; data from https://m.tianqi.com/lishi/heshun/201601.html	Binary	Camera trap
Moonlight Risk Index (MRI)	We calculated MRI by multiplying the percentage of the moon illuminated, the proportion of time between sunset and sunrise that the moon was above the horizon, and the proportion of the sky covered in clouds between 0 (overcast) and 1 (clear)	Continuous	Camera trap
Lunar phase	The moon phase was scaled to radians so that 0 relates to New Moon, First Quarter = π/2, Full Moon = π, and Last Quarter = 3π/2 for each species	Continuous	Moonrise
Anthropogenic Covariates			
Villages	Distance to the edge of the nearest human settlement	Continuous	(m)
Roads	Distance to the nearest road, including secondary road (small road) and tertiary road (dirt road or logging road); primary roads were excluded	Continuous	(m)

#### 2.2.2 Influence of moonlight on temporal overlap intensity

**FIGURE 2:**
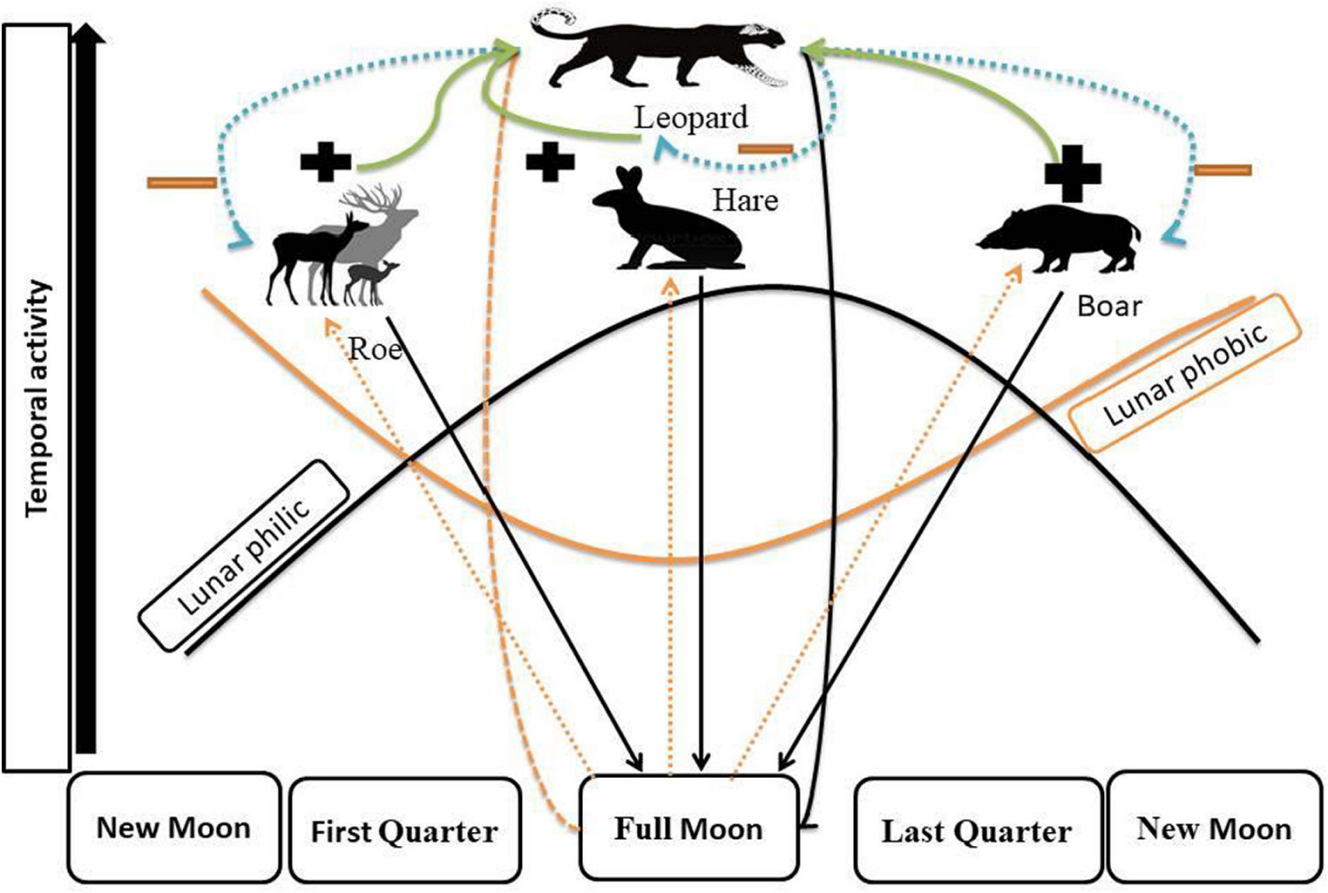
A conceptual diagram to explain that the predation risk hypothesis predicts that prey species will shrink activity during bright lunar illumination, denoted by orange color (lunar phobic), while the visual acuity hypothesis supposes that prey species which have comparably good vision will increase activity during bright lunar illumination, represented by black color (lunar philic). Those species positively linked are denoted by green solid lines, and negative interactions are represented by blue dotted lines

We calculated the Relative Abundance Index (RAI) of every species at each trap site as the number of detections per 100 camera‐trap days in the two seasons (Yang et al., 2019) (Table 2). Each camera trap was considered as an independent spatial point for determining animal nocturnal activity by selecting records occurring between sunset and sunrise. The clock time of sunrise and sunset varies slightly over the course of the year depending on the distance from the equator and time of year. To account for these successive changes in daylight hours throughout the year (Nouvellet et al., 2012), we used the “sunTime” function of the “overlap” package version 0.3.2 in R to map times to radians for analysis (see [Meredith & Ridout, 2020] for details). The activity pattern of each species was fitted nonparametrically as kernel density functions with the package “Overlap” using the default bandwidth parameters (Meredith & Ridout, 2014, 2020), following the assumption that animals are equally likely to be “trapped” throughout any period of their activity (Linkie & Ridout, 2011). Circular density curves were compared using the coefficient of overlap (“overlap coefficient”∆4), with values ranging from 0 (no overlap) to 1 (complete overlap), as proposed by (Ridout & Linkie, 2009). The lunar activity was categorized using Moonrise 3.5 software (https://moonrise.informer.com) to obtain the moon phase for each observation based on moon phase data stamped on camera trap images, location of the study area, and image/video data. The moon phase was scaled to radians so that 0 corresponds to New Moon, π/2 as First Quarter, π as Full Moon, and 3π/2 as the Last Quarter, as described by (Pratas‐Santiago et al., 2017; Prugh & Golden, 2014); further details depicted in the conceptual model (Figure 2).

**TABLE 2:** Seasonal number of photographic capture events and relative abundance index (RAI per 100 camera trap days) for the North China leopard and prey species as well as human activity (human on foot) in Tieqiaoshan Provincial Nature Reserve, Shanxi Province, China, from March 2017 to May 2019

Common name	Scientific name	Winter RAI	Summer RAI
North China leopard	*Panthera pardus japonensis*	117 (3.84)	37 (0.80)
Wild boar	*Sus scrofa*	288 (6.26)	126 (2.73)
Siberian roe deer	*Capreolus pygargus*	615 (13.37)	327 (7.11)
Tolai hare	*Lepus tolai*	1073 (23.33)	678 (14.74)
Human activity	*Homo sapiens*	768(16.69)	510(11.08)
Seasonal total	—	2921 (63.51)	1678 (36.48)
Study total	—	4599	

We estimated the overlap coefficient (∆4) for nocturnal activity between leopard–prey species pairs and night versus daytime for each species sets in four moon phases. In addition, to test the predictions of the predation risk and visual acuity hypotheses, we computed the number of independent records in each quarter of the moon phase for each species at night by dividing the moon phase cycle from 0 to 2π using four identical quadrants centered on every moon phase (for illustration, e.g., First Quarter from 1/4 π to 3/4π). The percentage of records in each lunar phase for each species was calculated, with the assumption that the lack of any pattern would be signaled by 25% of activity occurring during each of the four lunar phases (i.e., uniformity across moon phases). A deviation from 25% activity during the full moon phase was interpreted as follows: (1) species with less than or equal to 20% of records during the full moon were classified as lunar phobic; (2) those with more than or equal to 30% of records during full moon were considered lunar philic (e.g., Figure 2), and; (3) species that did not qualify as lunar phobic or lunar philic exhibited no pattern or irregular (Prugh & Golden, 2014).

#### 2.2.3 Model selection

To investigate potential factors influencing activity patterns associated with the lunar cycle, we developed two models: a mixed effects model to explore factors that might influence activity during the lunar month, and a logistic mixed effects model to examine whether the lunar cycle influences habitat preference or avoidance by leopard and prey during the lunar cycle.

For the first model that explored lunar cycle activity, we examined which factors were associated with an increase or decrease in activity closer to the full moon phase (e.g., lunar phobic versus lunar philic). To investigate this hypothesis, we used a Generalized Linear Mixed Effects Model (GLMM) using only nocturnal data (activity predominantly between 1 h after sunset and 1 h before sunrise), with temporal activity events occurring during the full moon as the response variable, checking whether lunar phase influenced animals' activities (Norris et al., 2010). We also categorized seasons as either summer or winter as we suspected that predators and prey may shift their activity in the different seasons (Yang et al., 2019). In addition, there is an ecologically significant factor which positively influences an animal's night and day activity called “Moonlight Risk Index” (MRI) (Gigliotti & Diefenbach, 2018; Searle et al., 2021). We calculated this index of nocturnal luminosity by multiplying together the amount of the moon illuminated, the proportion of time between sunset and sunrise that the moon was above the horizon, and the inverse proportion of the sky covered in clouds between 0 (overcast) and 1 (clear); moon‐based information was taken from moonrise software for each moon phase based on data stamped on camera trap images, and cloud cover information data for each capture event was taken from a free online data source in the Heshun county in TPNR (https://m.tianqi.com/lishi/heshun/201601.html)—more details provided in Table 1.

We tested the effects of the lunar cycle on leopard and prey activity in more detail by calculating circular statistics in the program Oriana (Bhatt et al., 2021). We allocated each observation night a numerical value, calculated as the days since the new moon divided by 29.5 (where 0 represents the new moon, and the number of days in the lunar cycle is 29.5). Then, we multiplied the results by 360°, such that 0° and 360° = new moon, and 180° = full moon (Bhatt et al., 2021). We used circular–linear correlation as the selected method of circular statistics (Mardia & Jupp, 2000), providing the mean vector (μ) and the length of the mean vector (r), where (r) is a measure of angular dispersion (related to standard deviation) and its value ranges from 0 to 1. In our study, a high r‐value indicates that animal activity is restricted to a specific lunar phase, whereas a low r‐value shows that activity is distributed across the lunar cycle. Rao's spacing test (U) for uniformity around the circular space was used to measure whether the animal activity was uniform across the lunar cycle (Bhatt et al., 2021). Rao's test is comparably more powerful and robust than many other circular goodness‐of‐fit tests and is capable to analyze bimodal and multimodal distributions, whereas other tests, such as the Rayleigh test and Watson's U2, cannot (Bergin, 1991).

First, for all global models in models 1 and 2, we checked for multicollinearity using the variance inflation factor (VIF) and Pearson correlation test, with covariates eliminated from our model at VIF >3 and/or |r| > 0.7 with other covariates (Yang et al., 2019). Second, for the coefficient of overlap, which is purely descriptive, we ran the Mardia–Watson–Wheeler (MWW) test ((Bhatt et al., 2021) to statistically relate the distribution of detections across the diel phase for leopard, prey, and human activity pairs (Bhatt et al., 2021; Botts et al., 2020) in program Oriana (Bhatt et al., 2021). If the value of W was higher than the critical value indicated at *p* < .05, we rejected the null hypothesis (Pewsey et al., 2013). Third, the GLMMs were fitted using the R package lme 4 and MuMIn (Yang et al., 2019). We used stepAIC to select the most parsimonious model at delta AICc ≤2 (Zaman et al., 2019; Zaman, Tolhurst, et al., 2020).

For the second model which explored circadian activity events during the four different lunar phases for leopard and prey linked to habitat factors, we considered activity occurrence events of the leopards and their prey species in each of the four moon phases during the night and daytime, classifying into the following three categories: (1) nocturnal, as defined above; (2) diurnal, activity predominantly between 1 h after sunrise and 1 h before sunset, and; (3) crepuscular, 1 h before sunrise to 1 h after sunrise, and 1 h before sunset to 1 h after sunset (Zhao et al., 2020). For example, if a species was less active during the full moon or other lunar phases at the nighttime (lunar phobic), we examined whether that species shifted to being more active during daylight (full moon day or other moon phases) hours to compensate for the time “lost” by being less active at night during the full moon phase. For each activity during night versus day events, ROC was used to define the accuracy of a classification model at the user‐defined threshold value of 0.5 (Zaman, Tolhurst, et al., 2020) as well as accepting models where the area under the curve (AUC) score was ≥0.7 (Jiang et al., 2015); we also ran a Shapiro–Wilk test and visual examination of histograms to confirm that the data were normally distributed.

In these second groups of models, we used a GLMM with the designation of day or night activity set as the binary response variable, irrespective of moon phase, where 0 represented daytime during all moon phases, and 1 represented nighttime during all moon phases, enabling us to compare day versus night activity. Habitat factors were included as explanatory variables and the camera trap identity was considered a random effect in our model to differentiate between the effects of non‐dependent variables, removing variables when a random effect was not found. We used nine covariates and ran seven GLMM candidate models to predict how these habitat factors altered species occurrence during the day versus night (Yang et al., 2019; Zaman et al., 2019; Zaman, Tolhurst, et al., 2020). Packages rocr, lmtest, car, lme 4, and MuMIn were used for GLMM analyses, and all analyses were computed in R statistical software V.3.5.1 (www.r‐project.org, R Core Development Team, 2018).

## 3 RESULTS

In this study's analyses, we used 102 camera traps for 4599 trap nights. We captured 154 events of leopards, 414 of wild boar (*Sus scrofa*), 942 of roe deer (*Capreolus pygargus*), 1751 of tolai hare (*Lepus tolai*), and 1278 events of human activity. The RAI for North China leopards in the summer (0.80) was lower than in winter (3.84), a phenomenon observed in all four species. Moreover, among the three main prey species, the RAI was highest for tolai hare and human activity in both seasons (Table 2).

### 3.1 Influence of moon phase on activity and predator–prey interaction

**FIGURE 3:**
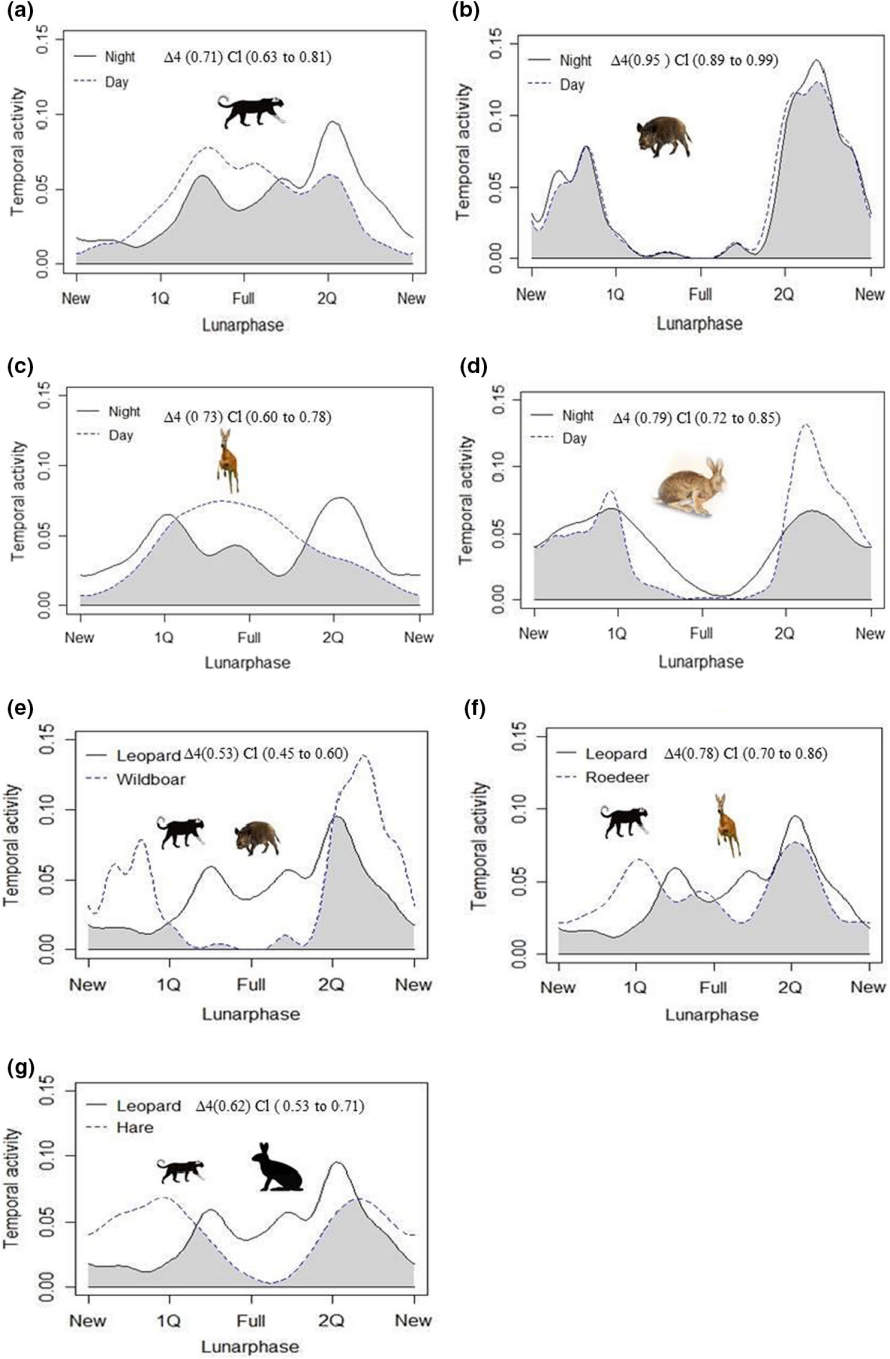
Temporal activity patterns of leopard (a, e–g), wild boar (b, e), roe deer (c, f), and tolai hare (d, g), including diurnal and nocturnal activity intensity (a–d) and overlap of predator–prey nocturnal activity (e and f). Wild boar and tolai hare distinctly reduce nocturnal and diurnal activity during the full moon, while leopard and roe deer increase diurnal activity during the full moon. High nocturnal activity overlap is observed between leopard and roe deer during the full lunar cycle and low overlap is observed between leopard and wild boar. Lunar phases are new moon (new), first quarter (1Q), full moon (full), and last quarter (2Q). In each plot e – g, temporal activity density is denoted by the solid line for predators (leopard) and broken line for prey species; gray shading represents the area of overlap using the coefficient of overlap (‘overlap coefficient’∆4) and CI (Confidence Intervals)

Leopards exhibited an irregular activity pattern and most camera trigger events occurred in the last quarter and full moon phase (Figure 3a; Table S2) during the nighttime, while daytime camera trigger occasions mostly occurred during the full moon phase and were fairly evenly spread during the other lunar phases. Overall, leopard activity was uniform during the lunar cycle (U = 321.82, *p* = .62) and was statistically more likely around the full moon (μ = 24.87°, *r* = .55) and first quarter (μ = 20.32°, *r* = .05).

For prey species, wild boar indicated lunar phobic behavior during the night; activity declined during the full moon, while peak activity occurred during the last quarter, followed by the new moon phase (Figure 3b; Table S2); similar results were found during the daytime, in which activity dropped during the full moon (Table S2) and were more consistent across other lunar phases (Figure 3b). Circular statistics showed that the activity of wild boar was non‐uniform during the lunar cycle (U = 336.30, *p* < .001) and was more active around the last quarter (μ = 133.33°, *r* = 0.61) and new moon (μ = 70.43°, *r* = .20). Roe deer were evidently lunar philic, based on percent nocturnal activity during the full moon phase and first quarter moon (Figure 3c; Table S2); daytime activity peaked during the full moon phase and was also relatively high during the first quarter moon and lowest during the new moon phase during both daytime and nighttime (Table S2). Furthermore, the circular statistics indicated temporal activity of roe deer was non‐uniform during the lunar cycle (U = 219.43, *p* = .001); roe deer increased their activity around the full moon (μ = 147.29°, *r* = .81) and decreased activity during the new moon (μ = 22.88°, *r* = .10). Human activities also occurred in the daytime more than nighttime and dawn/dusk (Table S2). Finally, similar to wild boar, tolai hare (Figure 3d) had a dip in nighttime activity during the full moon and increased activity during the first quarter (Figure 3d; Table S2); tolai hare daytime activity peak dipped in activity during the full moon and showed lunar phobic activity, increasing activity during of new moon (Figure 3d; Table S2). Furthermore, the circular statistics showed that the temporal activity of tolai hare was non‐uniform for the duration of the lunar cycle (U = 287.18, *p* < .001); tolai hare showed less activity around the full moon (μ = 22.89°, *r* = .44) and increased activity during the first quarter (μ = 182.87°, *r* = .10).

Each diurnal and nocturnal species pairing showed significant changes in circadian activities. The nocturnal temporal overlap between leopards and wild boar (Figure 3e) was low and the MWW test indicated non‐significant similarity between their diel activity shapes (W = 33.28, *p* = .41) (Figure 3e). In contrast, the temporal overlap between leopard and roe deer was relatively high and the MWW test indicated a significant relationship between their daily activity (W = 11.04, *p* < .01) (Figure 3f). Leopard temporal activity also exhibited comparatively high overlap with tolai hare and the MWW test indicated significant similarity between their diel activity shapes (W = 21.50, *p* < .01) (Figure 3g). The temporal activity of humans and both leopards (W = 33.40, *p* < .01) and wild boar (W = 18.10, *p* < .03) were statistically different. Conversely, the temporal activity of humans and roe deer (W = 31.20, *p* = .61) and tolai hare (W = 56.28, *p* = .81) were not statistically different in their diel activity patterns.

Based on analyses of nocturnal activity capture occasions during the full moon, leopard activity was significantly positively linked with MRI as well as the interaction between cloud cover and season (more active during a clear summer full moon) (Table 3). For roe deer, cloud cover at night did not influence temporal activity, though they were more active during a summer full moon than a winter full moon (a significant negative effect of season on activity during the full moon). Wild boar activity during the full moon was not influenced by cloud cover or MRI as independent factors, but their interaction had a significant negative effect. Finally, tolai hare activity during the full moon had a significant negative relationship with cloud cover (higher activity during clear nights) and a significant positive relationship with season (higher activity during summer).

**TABLE 3:** Results of the generalized linear mixed effects model of nocturnal activity capture occasions during the full moon from March 2017 to May 2019, including relation terms. (a) Leopard, (b) roe deer, (c) wild boar, (d) tolai hare

Variables	*Β*	SE	*z* value	*p*
(a) (Intercept)	0.69	0.06	11.29	.00
Clouds (clear, zero cloud)	0.06	0.10	0.60	.54
Seasons (summer)	0.01	0.06	0.26	.79
MRI	7.43	3.84	1.93	.05
Clouds×seasons	−0.28	0.11	−2.55	.01
Clouds ×MRI	−12.72	8.39	−1.51	.13
(b) (Intercept)	0.55	0.11	4.89	.00
Clouds (clear, zero cloud)	0.02	0.20	0.01	.99
Seasons (summer)	−0.22	0.09	−2.34	.02
MRI	6.91	6.35	1.08	.28
Clouds×seasons	0.04	0.16	0.25	.80
Clouds ×MRI	2.17	14.56	0.15	.88
(c) (Intercept)	0.58	0.13	4.39	.00
Clouds (clear, zero cloud)	0.30	0.18	1.60	.11
Seasons (summer)	0.17	0.11	1.43	.15
MRI	0.18	1.79	0.10	.91
Clouds × MRI	−5.95	2.80	−2.12	.03
(d) (Intercept)	0.55	0.04	11.34	.00
Clouds (clear, zero cloud)	−0.27	0.07	−3.78	.02
Seasons (summer)	0.17	0.07	2.39	.01
MRI	0.20	0.50	0.41	.67
Clouds×seasons	0.22	0.11	1.94	.05

### 3.2 Influence of habitat factors on species detections at day versus night

We found that leopards used lower elevations at night and higher elevations during the day, while activity closer to secondary roads and deciduous forests was more likely to be during the daytime and distances from these landscape features were more likely to be greater at night (Tables 4a and 4b). Roe deer also exhibited this relationship with distance to secondary roads in their day versus night activity analyses, although the relationship was weaker for roe deer. As observed in the leopard data, wild boar used lower elevations during the nighttime and medium or high elevations during the daytime; the relationship was stronger in the case of wild boar. Also, wild boar tended to be nearer to mixed forest during the daytime and further away at night. Finally, the tolai hare had a similar relationship with elevation as did leopard (i.e, weak negative association with elevation; lower elevations preferred at night), and at night tended to be far from mixed forest and grasslands while near deciduous forest, woody savannas, and villages.

**TABLE 4a:** Summary of generalized linear mixed effects candidate models (∆AIC <2) examining the influence of habitat factors on diurnal vs. nocturnal activity of leopard (a) and prey species (roe deer, b; wild boar, c; tolai hare, d). Data pooled from all moon phases. AIC_C_ = Akaike's information criterion adjusted for small sample sizes; K = degrees of freedom; Wi = Akaike weight

Model (explanatory variables)	*k*	AICc	∆AICc	*W*_*i*_
(a) Leopard ~ Elevation + distance to deciduous forest + distance to secondary road + distance to Tertiary road	5	265.02	00.00	0.63
Leopard ~ Elevation + distance to deciduous forest + distance to woody savannas + distance to secondary road + distance to tertiary road	6	267.01	00.02	0.23
(b) Roe ~ distance to deciduous forest + distance to secondary road + distance to tertiary road	4	215.64	00.00	1.75
(c) Boar ~ Elevation + distance to deciduous forest + distance to mixed forest	4	239.38	00.00	0.53
Boar ~ Elevation + distance to deciduous forest + distance to mixed forest + distance to secondary road	5	240.62	01.24	0.28
(d) Hare ~ Elevation + distance to deciduous forest + distance to mixed forest + distance to woody savannas + distance to grassland + distance to villages + distance to secondary road	8	383.79	00.00	0.72
Hare ~ Elevation + distance to deciduous forest + distance to mixed forest + distance to woody savannas + distance to grassland + distance to villages + distance to secondary road + distance to tertiary road	9	385.72	01.92	0.27

**TABLE 4b:** Parameter estimates of most parsimonious (best fitting) models in Table 3a for leopard (a), roe deer (b), wild boar (c), and tolai hare (d), including respective standard error (SE) and 95% confidence intervals. Asterisk (*) indicates model parameters with a significant effect on diurnal vs. nocturnal activity events linked to habitat factors. Data pooled for all moon phases

Parameter	Estimate	SE	95% CI
(a) Intercept	7.38	2.24	1.51 to 2.18
Elevation*	−0.46	1.53	−0.03 to ‐ 0.35
Distance to deciduous forest*	0.28	0.50	0.02 to 3.35
Distance to secondary road*	2.10	1.05	1.69 to 2.10
Distance to tertiary road	−0.02	0.04	−0.95 to 0.46
(b) Intercept	1.10	2.41	0.16 to 0.31
Distance to deciduous forest	−0.21	0.60	−7.99 to 1.74
Distance to secondary road*	0.01	0.03	0.48 to 0.78
Distance to tertiary road	−2.21	2.95	−7.45 to 0.24
(c) Intercept	3.46	3.81	2.78 to 4.29
Elevation*	−2.35	2.56	−2.90 to −1.89
Distance to deciduous forest	−0.79	2.80	−0.56 to 1.95
Distance to mixed forest*	0.73	0.90	0.24 to 0.29
(d) Intercept	14.16	2.05	10.26 to 18.32
Elevation*	−0.09	0.02	−0.07 to −0.01
Distance to deciduous forest*	−0.12	1.18	−0.92 to −0.70
Distance to mixed forest*	1.62	2.25	0.42 to 1.73
Distance to woody savannas*	−2.28	3.22	−0.83 to −0.59
Distance to grassland*	0.17	2.64	0.03 to 0.55
Distance to villages*	−1.25	1.54	−1.24 to −0.40
Distance to secondary road	−5.01	1.14	−0.03 to 4.70

## 4 DISCUSSION

We investigated the temporal ecology of predators and their prey, and their interactions in a human‐dominated landscape, Tieqiaoshan Nature Reserve, Shanxi province, China, providing baseline evidence of mammalian activity patterns, temporal niche segregation, and responses to the lunar cycle and habitat variables. In doing so, we have achieved our goal to better understand lunar effects on animal behavior, presenting novel insights on activity patterns in forested habitats, and, determining how leopards and prey species overlap or avoid each other temporally.

### 4.1 Influence of moon phase on temporal activity and predator–prey overlap

It is recognized that the response of nocturnal mammals to moonlight differs among taxa and may vary according to several determining factors, such as phylogeny, trophic level, sensory systems, and human activity (Santos et al., 2019). This camera‐trap‐based assessment has revealed both lunar phobic and lunar philic behaviors of rare and/or shy animals and contributes to building our understanding of the factors shaping activity patterns of multiple sympatric mammals under different lunar phases.

Our results indicated that leopards show no uniform activity pattern throughout the four lunar phases, exhibiting neither a clear lunar phobic nor lunar philic activity pattern, as observed in other felid species too, such as puma (*Puma concolor*) and jaguar (*Panthera onca*) in Neotropical forests (Harmsen et al., 2011; Prugh & Golden, 2014). Due to the extraordinary reflectance of the feline tapetum cellulosum, reportedly reflecting 130 times extra light than the human eye (Huck et al., 2017), we suspect that the leopards' lack of tendency to be more or less active during the full moon can be at least partly explained by visual acuity. We also suppose that the leopards' irregular temporal patterns may be influenced by mesopredators in the landscape, such as red fox (*Vulpes vulpes*) and leopard cat (*Prionailurus bengalensis*), as sympatric carnivores can drive temporal activity fluctuations (Hua et al., 2020; Packer et al., 2011). In terms of human activities, human activity on foot and in vehicles mostly occurred in the daytime in TPNR and human‐leopard conflict has previously been found to occur more often in the summer in TPNR when free‐roaming cattle grazing is more common (Hua et al., 2020). Perhaps, a landscape of fear induced by human activities may adjust leopard behavior (Lamichhane et al., 2019) and we suspect these phenomena may explain the irregular activity patterns and the higher relative species abundance in winter.

For the herbivore prey species studied, our results are similar to previous research showing that mammals often adapt their nocturnal activity to the level of lunar illumination (e.g., Huck et al., 2017, Pratas‐Santiago et al., 2017). Here, wild boar and tolai hare were evidently lunar phobic, while roe deer indicated lunar philic activity. Especially in the case of the wild boar and tolai hare, the results align with the visual acuity hypothesis, as lagomorphs and suids have poor eyesight and avoid the brightness of the moon to reduce predation risk (Pratas‐Santiago et al., 2017; Preisser et al., 2005; Tuan, 1979; Zaman, Rakha, et al., 2020). Lunar phobic behavior has also been observed in Neotropical prey species, such as armadillo (*Dasypus novemcinctus*) and paca (*Cuniculus paca*) (Harmsen et al., 2011, Prugh & Golden, 2014), while hare has also been observed to reduce the risk of predation by medium‐sized felids by adjusting temporal activity (Griffin et al., 2005). Deer species, however, including white‐tailed deer (*Odocoileus virginianus*) and red brocket deer (*Mazama temama*), have shown both lunar phobic and lunar philic behaviors, and while they have tapetum, prey can shift their night activity because the choroidal tapetum fibrosum (CTC) of carnivores has better light reflectance than the choroidal tapetum fibrosum (CTF) of ungulate herbivores (Botts et al., 2020; Brown et al., 1999).

Although felid activity patterns rarely relate to those of their potential prey (Ramesh et al., 2012) (Krittika & Yadav, 2020), in this study, leopard nocturnal activity had a high overlap with that of roe deer and tolai hare across the full lunar cycle. These results support the prediction that predators should reduce activity at times when major prey species are less active, in accordance with both the visual acuity and optimal foraging model (MacArthur & Pianka, 1966; Pianka, 1973). Conversely, prey can adjust their activity pattern or spatial distribution to avoid predators (i.e., to reduce predation risk), such as bighorn sheep (*Ovis canadensis*) spatially eluding pumas (*Puma concolor*) (Laundré et al., 2001), and roe deer and wild boar having minimal temporal overlap with tigers (Yang et al., 2019). Hence, while lunar phobia (wild boar and tolai hare) and lunar philia (roe deer) were observed in the prey species in this study, the activity pattern of leopards observed here, we suspect, is more closely linked with the temporal behaviors of prey species than directly with moon phase per se. Human activities also significantly influenced leopards and wild boar but had less effect on roe deer and tolai hare activity. Other studies also revealed that leopards avoided human settlements, and wild boar are considered a pest animal or otherwise are negatively perceived by people including because of crop damage (Zhou et al., 2021; Zhu et al., 2021); wild boar may, therefore, adopt spatiotemporal avoidance behaviors. However, species interactions and activities may be context specific as others have found that ungulate herbivores during daytime overlap with humans on foot in different habitats (Yang et al., 2021).

Explicitly during the full moon phase, nocturnal activity of leopards and prey were diversely affected by clear summer nights and moon risk index (MRI). Leopards' activity during the full moon showed a preference for high lunar luminosity (high MRI) and showed higher activity during clear summer or winter nights. We suppose that clear summer or winter nights or active prey may either force leopards to restrict roaming behavior (and reduce hunting success) or may increase activity as they move towards a safe or warmer refuge (Türk & Arnold, 1988). For prey species, nocturnal activity events during the full moon were more for roe deer in summer or winter (seasonal effect), during clear bright nights for wild boar (clear cover and MRI interaction effect), and during clear nights (especially summer) for tolai hare (clear cover and MRI interaction effects). Others have also revealed that cloud cover, which alters moonlight and MRI (Packer et al., 2011; Pratas‐Santiago et al., 2017) and season, influence nocturnality and temporal behaviors of carnivores and ungulates (Shamoon et al., 2018), as well as foraging behavior of wild boar and roe deer (Frauendorf et al., 2016; Yang, Dou, et al., 2018). It is reasonable to assume that there is variation in nocturnal activity between seasons.

### 4.2 Nighttime and daytime habitat use

Based on the GLMM model results, we revealed that leopard nocturnal and diurnal activity during the four moon phases changed in response to habitat variables. Specifically, during the daytime, leopards preferred to be closer to deciduous forests and secondary roads and to use areas of higher elevation (and vice versa for nighttime). We assume that these significant variables are associated with movement and hunting grounds for leopards at both day and night on a daily basis, offering higher concealment in deciduous forest, while undistributed roads provide substrate for territory marking (Macdonald et al., 2010) and also facilitate leopard travel (Carroll & Miquelle, 2006). Finally, in cat species, prey movement is the primary factor influencing habitat selection (Hayward et al., 2007). While we expect that leopards move throughout the fragmented landscape by using high elevation corridors, these highest areas also may provide suitable habitat and maximum hunting opportunity for ungulates or medium‐sized prey (Pu et al., 2019); the relationships with elevation were consistent across leopards and two of three prey species studied, while the leopards and roe deer had similar use of areas near to secondary roads during the daytime. (Yang et al., 2021; Zhu et al., 2021).

In addition to the results here derived from camera trap data, field observations (Zhu et al., 2021) and literature sources validate some of our conclusions concerning prey activity. For example, we encountered a great number of footprints of roe deer on (snow covered) secondary roads during winter field trips, as well as fecal pellets, footprints, and bedding sites of wild boar in mixed forest habitat. In the case of roe deer and secondary roads, we also observed that dogs used these roads and found that they may attack ungulates at nighttime, forcing these prey animals to temporally shift their use of the landscape to minimize risk (Young et al., 2018). With regard to mixed forests, Acevedo et al. (2006)) concluded that wild boar select broad‐leaved mixed forests as habitat, as these environments, dominated by Chinese red pine and Liaotung oak trees, provide foraging opportunities. While boar have been found to avoid high elevations at night (Roberts and Bernhard, 1977), and we may have suspected that this may be to avoid the predator vantage points at high elevation, leopard and boar showed similar trends in daytime/nighttime use, though we here offer a new insight into how elevation use may vary within a daily 24‐hour cycle. Finally, tolai hare showed a preference to be near open grassland and far from human settlement during the daytime; similar results have also been found for other lagomorph species in the Karakorum range, Pakistan (Zaman, Rakha, et al., 2020).

### 4.3 Implications for conservation

As daytime “super predators”, humans control 75% of Earth's land surface and are driving a rise in nocturnal activity of medium‐ and large‐bodied mammals (Clinchy et al., 2016). A recent meta‐analysis of studies of 62 mammalian species on six continents (Gaynor et al., 2018) exposed a robust influence of human existence on the temporal activity of wildlife, with an average 36% increase in nocturnal activity in response to human stimuli. The meta‐analysis revealed that nocturnal activity increased in response to an extensive range of human effects, lethal and nonlethal, including hunting, farming, and hiking; this proposes that wild animals recognize humans as dangers whether or not they pose a lethal risk (Gaynor et al., 2018). Free‐roaming dogs also cause both lethal and non‐lethal effects (Zaman et al., 2019). Even agricultural farming activities may cause mammals to shift to greater nocturnal behavior and reduced diurnal activity (Shamoon et al., 2018), and depredation on domestic livestock, which commonly occurs at night, can bring about negative perceptions and conservation issues (Mishra, 1997).

Using a variety of computational tools to analyze our vigorous dataset, this study has achieved two principal results which progress our understanding of the nocturnal and diurnal behavior of wild mammals in relation to the moon phase, including predator–prey interactions and effects of habitat factors. In particular, the study has revealed that wild animal patterns of activity across the study session display temporal flexibility in response to lunar illumination (as altered by moon phase, moonrise/set, cloud cover, night vision acuity (due to the tapetum lucidum, but not measured here), predation risk (as affected by the landscape factors plus the temporal overlap of a single predator), food availability (e.g., pine forest provides nuts), and potential competitive interference. Finally, recognizing that the North China leopard is a critically endangered species, to aid the conservation and management of this predator and its prey, we highly recommend further, deeper research on these focal species with a more integrated approach to understanding spatiotemporal patterns in response to natural and anthropogenic factors. We suggest that this research be aided by a live animal capture, collaring, and tracking protocol in combination with scientific evaluations of the top‐down and bottom‐up effects of human activities and land use, including roads, villages, and livestock farming. The results obtained from doing this research would likely help to scientifically—and optimally—manage the landscape for human–wildlife coexistence, restore wild animal communities and natural habitat, and increase landscape permeability and connectivity, enabling the flow of genetic material and long‐term population sustainability.

## AUTHOR CONTRIBUTIONS

**Nathan James Roberts:** Visualization (equal); writing – review and editing (equal). **Mengyan Zhu:** Investigation (equal). **Kasereka Vitekere:** Investigation (equal); methodology (equal). **Meng Wang:** Investigation (equal); methodology (equal). **Muhammad Zaman:** Conceptualization (equal); Visualization (equal); investigation (equal); methodology (equal). **Guangshun Jiang:** Conceptualization (equal); resources (equal); supervision (equal); validation (equal).

## CONFLICT OF INTEREST

The authors declare no conflict of interest.

## Supporting information


Figure S1
Table S1.
Table S2.

## Data Availability

The dataset (Table 1, S2 file) of this study is available upon request as Supplementary Material with a separate assignment.
