# Supplementary material for: Temporal activity patterns of North China leopards and their prey in response to moonlight and habitat factors
Source: Ecol Evol. 2022 Jun 23;12(6):e9032. doi: 10.1002/ece3.9032 (PMC9219109; doi:10.1002/ece3.9032)
Supplement: Supplementary file 1 — Figure S1 Table S1. Table S2. [file ECE3-12-e9032-s001.docx]

**
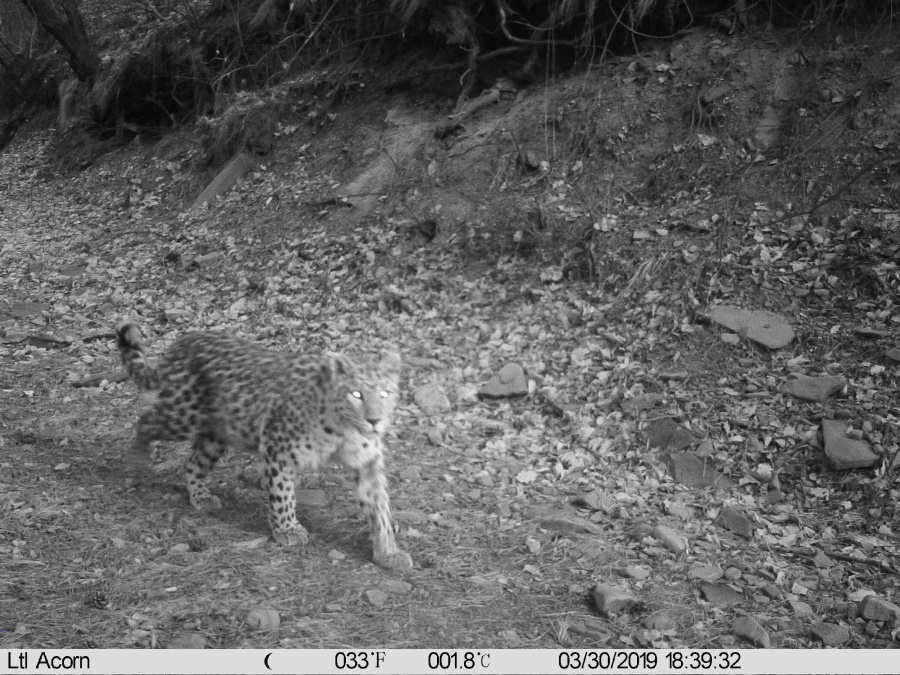
**

Figure s1: North china leopards camera trapping image in figure s1

**Table S1.** Worldwide food habits of leopard subspecies and respective prey consumption rates (percentages), as well as camera trapping studies of leopard-prey interactions.

| Leopards sub-species | Country | Method | Preferred prey | Prey consumed/interaction(%) | Status IUCN | References |
| --- | --- | --- | --- | --- | --- | --- |
| Sri Lankan Leopard *P .p .kotiya* | Sri Lanka | Diet analysis | Hare *Lepus nigricollis*  Wild Boar *Sus scrofa*  Barking Deer *Muntiacus muntjak* | 7.7  7.7  30.8 | Endangered | Kittle et al. 2014 |
| Indian leopards *P. p.fusca* | India | DNA analysis | Pig *Sus scrofa*  Sheep *Ovis aries*  Indian hare *Lepus nigricollis*  pig  *Sus scrofa*  Barking Deer *Muntiacus muntjak* | 0.6  0.8  14  11  13 | Endangered | Kshettry et al.2018  Desai et al. 2021 |
| Amur leopards *p. p .orientalis* | China | Scats analysis | Wild boar *Sus scrofa*  Roe deer *Capreolus pygargus*  Sika deer *Cervus Nippon*  Hare lupus species | 10.5  37.5  26.9  2.8 | Critically Endangered | Yang et al.,2018 |
| Persian Leopard  *P. p. saxicolor* | Iran | Scat analysis | Red Deer *Cervus elaphus maral*  Roe Deer  *Capreolus pygargus*  Wildboar *Sus scrofa* | 1.6  4.1  46.7 | Endangered | E. Sharbafi et al 2016 |
| Arabian leopards *P. p.*  *Nimr*  Indochinese leopard  *P. p.delacouri* | United  Arab Emirates  Cambodia | Scat analysis  DNA analyses | Ungulates: Goat hair and bone  Hare species lepus spp  Cape hare *Lepus capensis*  Banteng *Bos javanicus*  Wild pig  *Sus scrofa*  Leopard cat *Prionailurus bengalensis*  Burmese hare *Lepus peguensis* | 90.4  28.5  80  17.8  39.7  1.4  4.1 | Critically Endangered  Critically Endangered | Chris and Stuart,2017  Drew . 2000  García et al.,2018 |
| Snow leopard *Panthera uncia*  North China  leopard *P. p.japonensis* | Pakistan  China | Scat analysis  Camera trapping | Ibex *Capra ibex sibirica*  Markhor *Capra falconeri*  Cape hare *Lepus capensis*  Wild boar *Sus scrofa*  Roe deer *Capreolus pygargus*  Hare lepus spp | 2.4  4.8  4  22.5  24.3 | Endangered  Critically Endangered | Khatoon et al.,2017  H. Yang et al 2020 |
| African leopard *p.p.pardus* | Tanzania | DNA analysis | Small prey (<19 kg)  large prey (≥80 kg) | 70  50 | Critically Endangered | Havmøller et al 2021 |
| Javan leopard *P. p. melas* | Java | Camera trapping | Java mousedeer *Tragulus javanicus*  Red muntjac *Muntiacus muntjak*  Wildboar *Sus scrofa*  Banteng *Bos javanicus* | 0.1  0.3  –0.1  0.2 | Endangered | Rahman et al.2018 |

**Table S2.** The number of photographic events (and percentages) captured for leopards and prey during nocturnal/crespcular and diurnal activity periods with respect to the four distinguished moon phases in Tieqiaoshan nature reserve, China

|  | Full study | Nocturnal/crepuscular | | | | | Diurnal | | | | |
| --- | --- | --- | --- | --- | --- | --- | --- | --- | --- | --- | --- |
| Species | Total | Total | New moon | First quarter | Full moon | Last quarter | Total | New moon | First quarter | Full moon | Last quarter |
| North china leopard | 154 | 98(63.63) | 20(12.98) | 18(12.68) | 33(21.42) | 27(17.53) | 56(36.36) | 7(04.54) | 7(04.54) | 34(22.07) | 8(14.81) |
| Wild boar | 366 | 150(40.98) | 34(09.28) | 34(09.28) | 32(08.74) | 50(13.66) | 216(59.01) | 67(18.30) | 44(12.02) | 29(07.92) | 76(20.76) |
| Siberian Roe deer | 624 | 325(52.08) | 37(05.92) | 60(09.61) | 188(30.12) | 40(06.41) | 299(47.91) | 39(06.25) | 40(06.41) | 190(30.44) | 30(04.80) |
| Tolai hare | 1,241 | 595(47.94) | 115(12.48) | 193(15.55) | 103(08.29) | 144(11.60) | 646(52.05) | 187(15.06) | 127(10.23) | 122(09.83) | 210(16.92) |
| Human activity | 1,198 | 233(19.44) | - | - | - | - | 965(80.55) | - | - | - | - |
| Study total | 3,667 | 1,404(38.28) | 206(05.61) | 309(08.42) | 352(09.59) | 261(07.11) | 2,263(61.71) | 300(08.18) | 227(06.19) | 430(11.72) | 341(09.29) |
